# DiseaseNet: a transfer learning approach to noncommunicable disease classification

**DOI:** 10.1186/s12859-024-05734-5

**Published:** 2024-03-11

**Authors:** Steven Gore, Bailey Meche, Danyang Shao, Benjamin Ginnett, Kelly Zhou, Rajeev K. Azad

**Affiliations:** 1 Department of Biological Sciences and BioDiscovery Institute, University of North Texas, Denton, TX USA; 2 Department of Mathematics, University of Louisiana at Lafayette, Lafayette, LA USA; 3 Department of Engineering, Eastern Arizona College, Thatcher, AZ USA; 4 Department of Computer Science and Engineering, University of North Texas, Denton, TX USA

**Keywords:** Noncommunicable diseases, Classification, DNA methylation, Machine learning, Deep learning, Transfer learning

## Abstract

As noncommunicable diseases (NCDs) pose a significant global health burden, identifying effective diagnostic and predictive markers for these diseases is of paramount importance. Epigenetic modifications, such as DNA methylation, have emerged as potential indicators for NCDs. These have previously been exploited in other contexts within the framework of neural network models that capture complex relationships within the data. Applications of neural networks have led to significant breakthroughs in various biological or biomedical fields but these have not yet been effectively applied to NCD modeling. This is, in part, due to limited datasets that are not amenable to building of robust neural network models. In this work, we leveraged a neural network trained on one class of NCDs, cancer, as the basis for a transfer learning approach to non-cancer NCD modeling. Our results demonstrate promising performance of the model in predicting three NCDs, namely, arthritis, asthma, and schizophrenia, for the respective blood samples, with an overall accuracy (f-measure) of 94.5%. Furthermore, a concept based explanation method called Testing with Concept Activation Vectors (TCAV) was used to investigate the importance of the sample sources and understand how future training datasets for multiple NCD models may be improved. Our findings highlight the effectiveness of transfer learning in developing accurate diagnostic and predictive models for NCDs.

**Supplementary Information:**

The online version contains supplementary material available at 10.1186/s12859-024-05734-5.

## Introduction

Noncommunicable diseases (NCDs) are responsible for approximately 7 in 10 deaths worldwide, surpassing all deaths attributable to communicable diseases combined in 2021. The impact of NCDs is profound, with diseases such as asthma, arthritis, and schizophrenia (SCZ) posing significant threats. Even when the NCDs are not fatal or terminal, they contribute to a significant loss in the quality of life for affected individuals [1]. Asthma, arthritis, and SCZ are known to have devastating effects on the quality of life, even leading to premature death. Asthma is the most common NCD in children worldwide and is the cause of an estimated 455,000 deaths every year [2].

Ongoing efforts to understand the molecular underpinnings of NCDs have relied heavily on genetic data, which have often been leveraged by genome-scale methodologies, such as Genome-Wide Association Studies (GWAS), to identify genetic loci associated with these diseases. Epigenetics has more recently been implicated in the etiology and identification of NCDs [3–12]. DNA methylation that manifests prominently on the epigenetic landscape has gained attention in understanding the NCD etiology. In an effort to expand further on this, our study here attempts to address the challenges with limited data availability for many NCDs by introducing a novel approach—the utilization of transfer learning from a pre-built neural network model of pan-cancers. This approach allowed leveraging a wealth of data available for cancers and using it as a foundation for building robust neural network models for non-cancer NCDs. Here, we focused on interrogating a vast amount of publicly available DNA methylation data, previously established as a key component in disease classification, which were obtained from DNA methylation sites in the human genome. In the following sections, we delve into the broader landscape of NCDs, examining the role of methylation and its implications for disease detection. This exploration provides essential context for our subsequent discussion on the application of transfer learning and neural networks to model non-cancer NCDs.

The use of next-generation omics-based methods in these efforts has produced rich datasets that may be leveraged to develop powerful neural network based models of these NCDs. While growing, the volume of data available for many NCDs is still too low to train neural networks from the available data alone. However, large amount of omics and other data are available for some NCD families, such as cancer, which may allow the use of transfer learning to generate neural network models of NCDs that lack enough data to build such models. One such type of data of interest for disease classification is methylation data, procured from DNA methylation sites in the human genome. However, it is yet to be shown that model trained on methylation data from one class of NCD, such as cancer, can be used as the basis for models of other NCDs.

Machine learning models have been applied to many types of omics data to address a variety of biological questions [13–19]. Cancer has been a focal point for many researchers and the research in this domain has been powered by some of the largest and well-curated omics datasets available. Non-cancer NCDs, while actively and passionately researched, generally make up a much smaller share of the available disease omics data as of this publication. Due to this, model based NCD research is generally limited to a small set of features making it difficult to find interactions outside of those features. Additionally, models of non-cancer NCDs tend to be limited to the specific disease, or disease family, being researched rather than incorporation of multiple diseases within an integrated model. Ideally, emerging models should incorporate larger numbers of disease types and rely on larger feature sets.

We posit that NCDs exist on a landscape, and if it is so, then a model trained on one NCD, or NCD family, could be extended or retrained on another due to the transferability of information learned about one disease to another. Models such as this would allow researchers to find common risk factors or understand complex risk factors, which could aid in the discovery of biomarkers or development of novel therapies.

Previously, we had trained a model, CancerNet, on DNA methylation data [16]. CancerNet detected cancers and their tissues of origin using an integrative model of cancers encompassing 33 cancers represented in The Cancer Genome Atlas (TCGA), with a very high accuracy (over 99%). Further, CancerNet compared favorably with other models, including those based on probabilistic and ensemble learning methods [16]. Here, we use transfer learning to train the CancerNet model to identify 3 NCDs: Asthma, arthritis and SCZ. To our knowledge, no other models have been produced that incorporate multiple NCDs or non-cancer NCDs with cancer samples.

### Non-communicable diseases and methylation

Genome-Wide Association Studies (GWAS) have revealed genetic loci associated with asthma [8, 20, 21], arthritis [10, 11, 22–27], and SCZ [3–5, 9, 28–33], but these loci are responsible for a fraction of the risk [12]. Epigenetic studies involving gene expression, histone modifications, and methylation have revealed evidence that implicates epigenetic risk factors, which may work in concert with genetic risk factors [12, 18, 27, 34].

Each of the three diseases manifests in or affects different tissues. This could make modeling even more complicated as the model may learn tissue specific signatures rather than disease specific ones. We selected methylation data from peripheral blood samples as these were available for all three diseases we chose for this study; further previous studies have reported the prevalence of epigenetic signatures of these diseases in peripheral blood samples [3, 8, 24, 28, 30, 33, 35]. Additionally, early risk factors for SCZ were found to be prevalent in methylation of peripheral blood cells [29, 30].

Other than the large volume of data available for cancer, already known links between cancer and other NCDs, such as SCZ, provide evidence that there may be overlapping risk factors. This indicates that cancer may exist on the same (epi)genetic landscape as other non-cancer NCDs. It has been reported that methylation aberrations along with tumor suppressor regulatory changes appear to directly link SCZ with cancer rate [36]. The glucocorticoid receptor gene NR3C1 is also implicated in multiple neurological NCDs including SCZ [7] and is a predictor of poor prognosis in estrogen receptor (ER) breast cancer [37] Indeed, longitudinal studies with second generation antipsychotics show that their mechanism is strongly linked to renormalizing methylation changes associated with SCZ [32]. Links such as these serve to illustrate why the development of a pan NCD model through transfer learning is biologically feasible and likely to generate a clinically useful model as well as a rich, biologically meaningful, latent space from the data.

### Non-communicable disease detection with machine learning

Research that exploits differential NCD methylation pattern within a neural network framework often focuses on one disease or family of diseases [7, 10, 13–15, 17, 22, 26, 27, 38]. The datasets used in each such model are small, requiring heavy use of feature selection. This unnecessarily limits the scope of the model and limits the information learned by the model. Transfer learning that has been underutilized as a modeling tool in this field provides significant advantages in that the disease of interest may be understood in the context of other NCDs and within a richer information space.

Transfer learning is a machine learning technique that utilizes information learned by a model trained for one task on another task the model has not been explicitly trained for [39]. Generally, the two tasks are expected to be in a similar domain and share low level features [39–42]. Because the two tasks are related, the pretrained model is expected to have parameters much closer to those needed to perform well in the new task. This means the search space is greatly reduced and the new task may be learned with fewer examples [43].

Previously, a model trained on expression data, MultiPLIER, was successfully transferred to model other rare NCDs in the expression space [44]. This was done using an unsupervised PLIER network [19] and resulted in a model with a rich latent space. Our method differs mostly due to the use of methylation as our input data. Methylation is easily detected in blood due to longer half-life of DNA in circulating blood as compared to freely circulating RNA. Additionally, RNA is best assessed from a whole cell environment making it limited in its use as a diagnostic or predictive dataset in these cases. The methylome, however, is not as well characterized and has less prior information to rely on, making it a poor candidate for PLIER model training which requires prior information as input to the model. For the reasons mentioned above, we here sought to exploit DNA methylation data for NCD classification within a transfer learning framework.

To our knowledge, this is the first attempt to utilize transfer learning to train a multi non-cancer NCD model. This innovative approach allows multiple diseases to be studied in the context of other, possibly distantly related, human diseases. Indeed, our approach worked despite the remarkable differences between the NCDs and the cancers, the former selected for classification using a multi non-cancer NCD model and the latter for the transfer learning purpose using the trained CancerNet model. These promising results were rather unexpected and will spur further novel applications of transfer learning to disease classification and beyond in the biomedical field.

## Materials and methods

### Data and preprocessing

We obtained methylation datasets of NCDs (accession numbers: GSE36054, GSE41169, GSE56553, GSE69270, GSE71841, GSE89251, GSE99863, GSE111942, GSE121192, GSE152027, GSE174422) from the NCBI GEO database (https://www.ncbi.nlm.nih.gov/geo/). Among these, 659 samples represent Schizophrenia (SCZ), 72 samples are of Asthma, 203 samples correspond to Arthritis, and 1224 samples are of the Normal (Norm) class.

We used the MethylSuite package to download and prepare raw methylation data for each dataset. Each sample was put through our processing pipeline to generate 24,565 CpG (cytosine-guanine nucleotides) islands. These islands were determined using the method outlined in the CancerNet paper [16] and are as follows. When two CpGs were found within 100 base pairs of each other, they were grouped in a cluster. If a CpG already in a cluster was within 100 bp of another CpG, the new CpG was added to that cluster, i.e. the two clusters were merged. The average beta-value representing the methylation intensity was computed for each cluster and then used as the input. While this simple approach for clustering of CpGs works well [16], other approaches including deep clustering techniques [49] may also be explored.

### Model

**Fig. 1:**
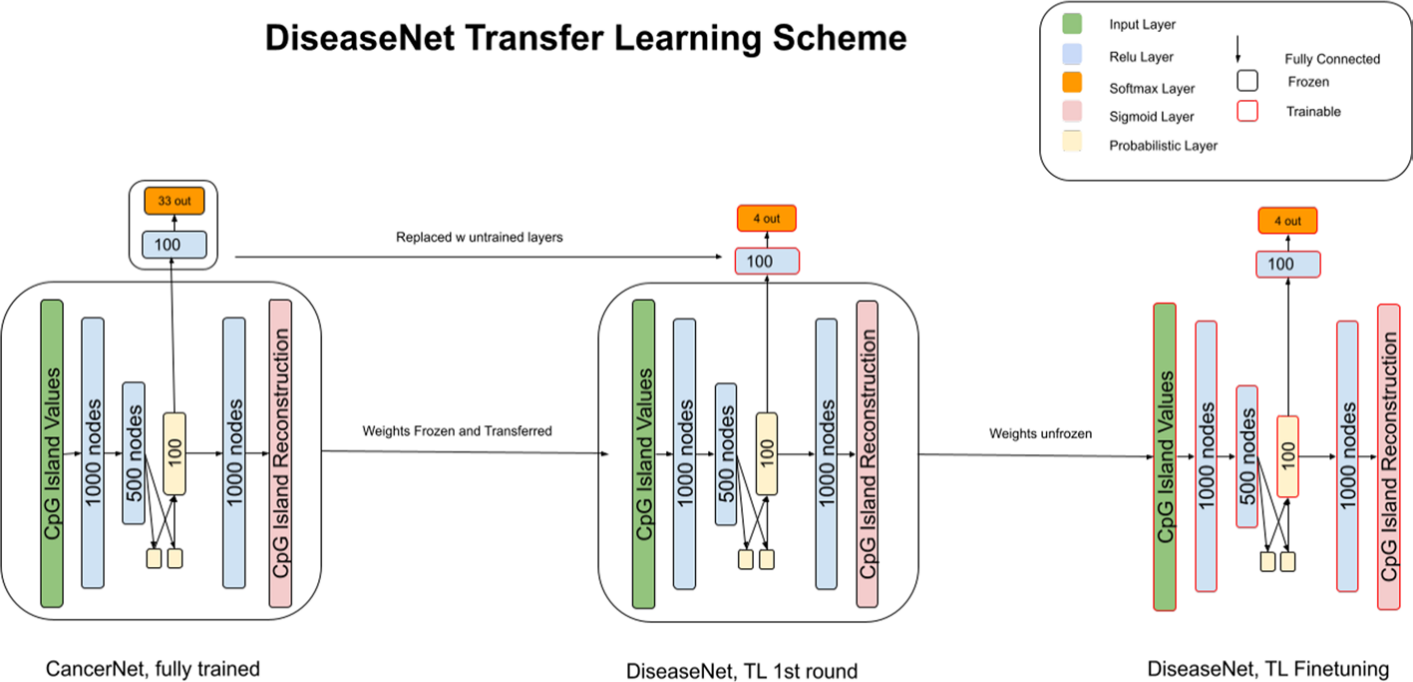
DiseaseNet architecture and transfer learning scheme. Left: Fully trained CancerNet. The transfer learning process starts with the fully trained CancerNet. Center: Transformation to DiseaseNet from CancerNet. The weights are frozen for the encoder and decoder. The classification layers (top) are replaced with layers with randomly initialized weights. The output (softmax) layer has four nodes representing the four diseases being classified. In the first round of training, only the classifier is trained. Right: Finetuning of DiseaseNet. Weights of the entire model are unfrozen and allowed to train in the last round until convergence

We used here the same model architecture as was used in CancerNet [16] which utilizes a variational autoencoder (VAE) that has a classification task trained at the same time as the generative task (Fig. 1). All layers are dense layers unless otherwise noted. The encoder has 24,565 input nodes. The input features are the mean beta values for 24,565 CpG islands that were calculated as described in the data preprocessing section. The encoder is made up of two hidden layers with 1000 and 500 nodes each. This is followed by a sampling layer made up of 100 nodes.

The sampling layer’s output is then used as input to a classifier and a decoder. The classifier has a 100-node layer followed by an output layer that has either 4 or 37 nodes for the transfer learning task or the retraining task respectively. The output layer uses a softmax activation function.

The decoder has a 1000-node layer followed by a 24,565-node output layer. The output of the decoder uses a sigmoid activation function. The loss function is the weighted sum of the classifier and decoder individual losses. The losses used are categorical cross entropy loss and VAE loss for the classifier and decoder, respectively. Weights of 1 and 0.001 were used for classifier and decoder, respectively. The model was implemented in Keras [45]; for a list of dependencies, refer to the readme file at https://github.com/Sgore83/DiseaseNet. For the transfer learning task, the model was initialized with the trained weights from CancerNet, while the full retrain was initialized with the initial random weights from CancerNet to minimize starting state variability.

### Transfer learning

The complete data set was divided into training, validation and test sets. To assess the robustness and generalizability of the model, we employed a tenfold cross-validation strategy. The dataset was partitioned into ten folds (subsets), with each fold used for test and the remaining data used for training (eightfold) and validation (onefold); the training with validation and test processes were thus repeated ten times and the performance metrics, described below, were computed as the average over the tenfold.

CancerNet’s architecture and weights were loaded and the final two layers of the classifier’s output were replaced by randomly initialized weights for the new classification output size of 4. We then froze the weights of the original layers and trained the last two classifier layers until convergence. Early stopping with a patience of 100 epochs was used to determine convergence. The weight file was only updated when the model improved.

Fine tuning was then done by making the whole model trainable and training the whole model with a learning rate of 1 × 10^−6^. This second round of training was allowed to train until convergence with an early stopping patience of 200 epochs. The weight file was only updated when the model improved.

### Binary classifier models

Binary model for each class (i.e. that class versus the rest) was trained using the same architecture as DiseaseNet except that the classifier output layer had a single binary node. For each class, samples were labeled using a positive (target class) versus negative scheme, where any sample not belonging to the target class was labeled as the negative class.

### Comparative assessment with other machine learning models

We conducted a comparative assessment by applying other machine learning models to the same dataset that DiseaseNet was subjected to. This comparative analysis involved well-established models such as Random Forest, Support Vector Machine, K-Nearest Neighbors, and Decision Tree. Each model was evaluated based on the performance metrics precision, recall, and F1 score (see the next section).

### Performance metrics

We assessed the model performance using F-measure, which is the harmonic mean of Precision and Recall define as follows.

$$Precision = \frac{TP}{{TP + FP}}$$

$$Recall = \frac{TP}{{TP + FN}}$$

$$F - measure = \frac{2 \times Precision \times Recall}{{Precision + Recall}}$$

Here TP is the count of true positives, FN is the count of false negatives, and FP is the count of false positives.

## Results

### Performance assessment of DiseaseNet

**Fig. 2:**
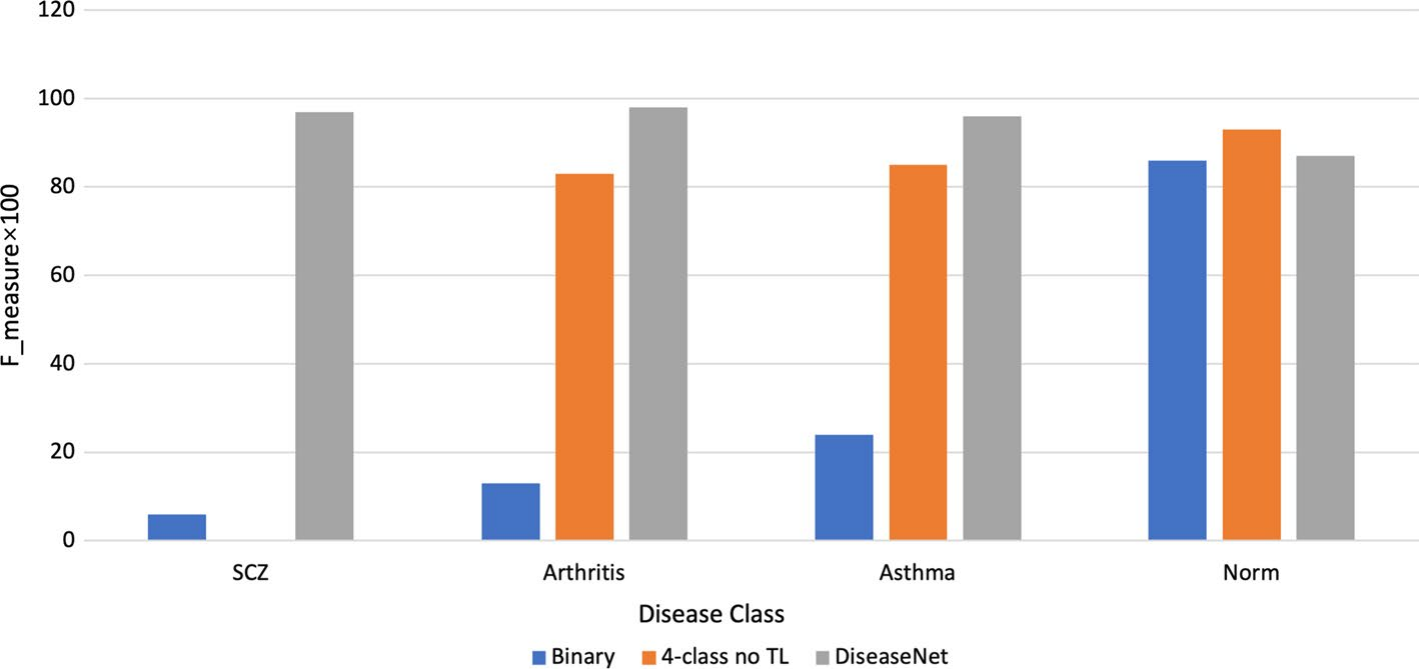
Comparison of models for different training schemes. For each disease class, the values of F-measure for the binary models, 4-class model without transfer learning, and fully trained DiseaseNet (with transfer learning), are shown by the blue, orange and grey bars respectively. Overall, DiseaseNet outperformed models representing different architectures and/or training options

Our approach to a robust NCD classification was based on the premise that a model trained on cancer methylation data contains information that could be leveraged to train a new model of non-cancer NCD based on the methylation data. With CancerNet as our initial model, we randomly initialized a new classification layer with 4 output nodes (SCZ, Arthritis, Asthma, and Normal) and trained only the new layer. We then unfroze the rest of the model and trained it with a very low learning rate. The resulting model, DiseaseNet, produced a classification F-measure of 94.5% on average and F-measure of 97%, 98%, 96%, and 87% for SCZ, Arthritis, Asthma, and Normal classes, respectively (Fig. 2).

We assessed the performance of other frequently used machine learning models on the same NCD dataset the DiseaseNet was evaluated on. DiseaseNet with an average F-measure of 94.5% compared favorably with the other models; Random Forest and Logistic Regression generated average F-measure of 0.88, followed by Support Vector Classifier, and Decision Tree, and K-Nearest Neighbors at 0.86, 0.82, and 0.79 respectively.

As a sanity check, we trained binary classifiers for each NCD (see Methods), excluding the step of transfer learning in this process. The performance of these individual models serves as a class specific lower bound that is derived from the information contained in the non-cancer NCD dataset only. If these models perform as well or better than the transfer learning approach, then we could attribute performance largely to the variation between individual NCD classes itself rather than to the transfer learning process. The F-measure values for the single NCD (and normal) class models—SZD, Asthma, Arthritis, and Normal models—were 6%, 13%, 24%, and 86%, respectively (Fig. 2). This indicates that in most classes, the transfer learning approach added significant information not previously contained in the non-cancer NCD data alone.

We then trained a 4-class NCD model without transfer learning. This served as a fine-grained test on the information overlap among classes in the non-cancer NCD dataset. The values of F-measure for the classes were—SCZ: 0%, Asthma: 83%, Arthritis: 85%, Normal: 93% (Fig. 2). Compared to the binary classification without transfer learning, large improvements were observed in all but one classes, which demonstrates there is contrastive information allowing classification of some classes to improve when the model is provided with this information. The F-measure value was highest for the normal and further, this was the highest overall accuracy achieved for the normal among the three scenarios considered here (binary without transfer learning, 4-class without transfer learning, and 4-class with transfer learning). Surprisingly, with 4-class without transfer learning, the model produced 0% overall accuracy (F-measure) for SCZ, that is, it failed to learn at all for this class. The overall performance of the model is likely due to learning a suboptimal weight set during model training that sacrifices SCZ performance for higher performance for the normal. Apparently, this also highlights the vagaries of usage of deep learning models when training data are scarce for one or more classes. High accuracies attained through transfer learning demonstrates its effectiveness in mitigating these issues with deep learning. Here, the transfer learning approach performs approximately as well as the lower bound for the normal class that was established with the binary classifier but improves in all other classes significantly. This indicates that the parameters from a model trained on cancer methylation data may be transferred to create an improved model of other non-cancer NCDs with less support.

### Model characterization

**Fig. 3:**
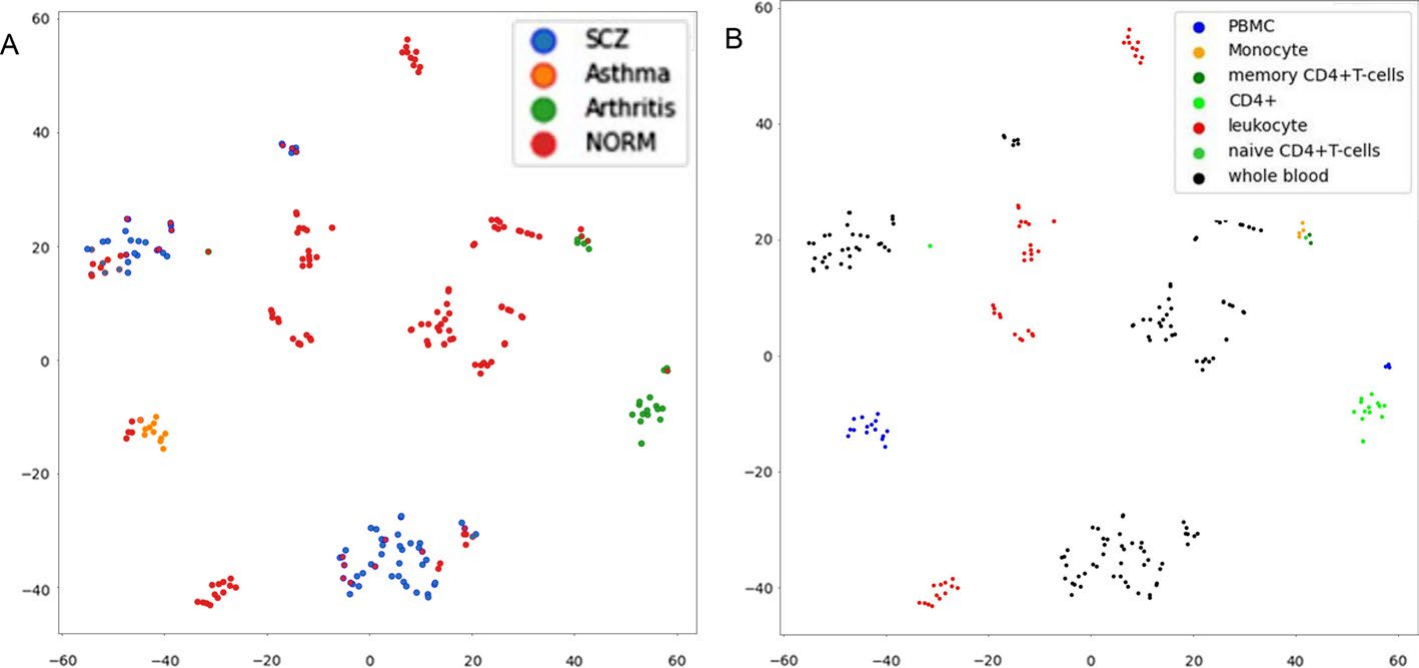
Distribution of Samples in DiseaseNet Latent Space. **A** Schizophrenia (SCZ), Asthma, Arthritis, and Normal (Norm) samples are color-coded with blue, yellow, green, and red respectively in the DiseaseNet’s latent space generated using t-SNE. They are well separated in the space with only a few places of overlap by class. **B** Samples are color-coded by their tissue source (sources shown in the legend). The latent space shows a clear separation by sources; this indicates that tissue sources play an important role in the classification of the diseases by this model

In our analysis, we employed t-distributed stochastic neighbor embedding (t-SNE) to visualize the latent space and examine the distribution of disease (and normal) classes (Fig. 3a). t-SNE is a powerful dimensionality reduction technique known for preserving local structures in high-dimensional data. Specifically, it maps high-dimensional data points to a lower-dimensional space while maintaining their pairwise similarities. Visual inspection of the t-SNE plot revealed disease segregation with some noticeable overlap between normal and SCZ samples, indicating the likely source of misclassifications by this model between these two classes. Further insights were provided by the source types of the datasets. Notably, the SCZ and asthma datasets were exclusively composed of a single source type, namely, the whole blood and the peripheral blood mononucleate cells (PBMCs), respectively, while the normal and arthritis datasets consisted of multiple cell types (Additional file 1: Table 1). This dataset composition variability may contribute to the observed patterns in the t-SNE visualization, emphasizing the importance of understanding the characteristics of the input data in the interpretation of classification results.

Inspecting the latent space based on the sample sources, we found that the source types segregated well and did not intermingle (Fig. 3b). This is not surprising as samples with different cellular makeup are expected to have differing methylomic signatures and should occupy different areas of the latent space. The primary question is how important the cell-specific signatures are to the classification output of the model.

To investigate this, we leveraged the fact that ‘cell type’ is encoded as a concept vector within DiseaseNet. Concepts are high level features of an input that may or may not be explicitly trained into the model. A class label in a classification task is a trained concept, though not the only one learned, whereas a generative model learns many concepts without the need for a label. They are represented within neural networks as vectors in some latent layer. These concept vectors can be detected, and an importance score assigned by a method called Testing with Concept Activation Vectors (TCAV) [46]. TCAV results indicate how significant a concept, cell source in this case, is to a given class.

TCAV scores for each class are provided in Additional file 1: Tables 2–5 and source distributions are provided in Additional file 1: Table 1. We found that classes have higher TCAV scores (importance) for sources from which they were derived, in general. An exception to this is the PBMC source. It has low importance in every class for which it occurs; arthritis, asthma and normal (Additional file 1: Tables 2–4). In contrast, when classes have samples from multiple sources, this does little to decrease the importance of the source concept, as can be observed in the arthritis TCAV scores where monocyte and CD4+ concepts have high TCAV scores, as opposed to the PBMC score which is low in this class (Additional file 1: Table 2). On the contrary, Asthma is the only class for which the only source is PBMC, however, PBMC has a low TCAV score for this class. Oddly, the CD4+ concept is highly important to the asthma class despite not being a source for asthma samples. The consistently low TCAV score for PBMC across all classes, with most classes being partly sourced from PBMC, demonstrates the tendency for confounding conceptual/contextual information to be minimized when stratified across classes.

## Discussion

Deep learning models have shown promising results when large amounts of training data are available for learning the model parameters; however, in some domains such as medicine, the training data could be scarce for certain problems including disease diagnosis. Lack of biomedical data limits the applicability of deep learning models to address problems of profound importance in medicine such as disease detection with precision. To circumvent this issue and unleash the power of deep learning in medicine, transfer learning is increasingly being exploited to leverage information underlying training data to the maximum extent possible, specifically in transferring information learnt robustly for certain problems in a domain to address different but related problems within the same domain. We posited that discriminatory methylation patterns in cancers learnt by CancerNet may provide useful information for non-cancer NCD classification as NCDs share the same domain. We, therefore, hypothesized that utilizing the pre-trained knowledge and feature representations of cancer methylation patterns deciphered by CancerNet is an efficient approach to the classification problem for NCDs where training data is a limiting factor for the use of deep learning models. Using transfer learning, we built a robust model for non-cancer NCD classification. This approach not only holds promise for improving individual disease prediction but also for revealing shared risk factors, identifying novel biomarkers, and generating pan-NCD models with insights into disease mechanisms.

This study demonstrates the effectiveness of transfer learning in the generation of generalizable NCD models. In particular we highlight the potential that such an approach has in improving modeling of NCDs with small support sets. DiseaseNet, as described with 4 classes here, is a proof of concept that may spur further applications of transfer leaning to disease diagnosis and classification. Increasing tissue sources and disease classes is the primary focus of our ongoing effort to model the NCD landscape further.

We also demonstrated the concept-based explanation of our model. It is important to note that high TCAV scores are indicative of greater importance of the concept to a class; there may be many concepts that are important. Individual concepts do not demonstrate completeness of the class concept. The TCAV results demonstrate that models such as ours may benefit from diverse tissue sources for different diseases. The implication here is that larger and more diverse datasets should be obtained, however, this is a known issue among many NCD studies and the retrieval of such datasets. Instead, we believe data augmentation of biological datasets would greatly improve such models without incurring high costs and efforts/time invested in gathering further data from wet lab experiments. Data augmentation has proven highly effective in model training in the field of computer vision and it stands to reason that it would positively benefit omics modeling as well.

The field of computer vision is easily understood by human vision and augmentations are obvious (such as rotation, cropping and brightness). Omics data, broadly, do not benefit from easy human interpretation and the primary issue here is the difficulty in understanding which augmentations would be most pertinent and how to apply them in an omics setting. As an example, our samples have 24,565 input features. Each is real-valued and many features are dependent upon others or at least correlated. It is difficult to understand how to change those samples so that we may augment a concept and minimally affect the information that is important. We suggest that concept vectors be used in a generative model, such as DiseaseNet, to create an augmented sample set. Such sample sets could be easily produced and would result in better models. The limitation is that the concept being augmented must be understood well and explained in the context. The explainability methods such as TCAV [46] and Shapley Additive exPlanations (SHAP) [47] may greatly benefit augmentation efforts.

The VAE portion of models such as DiseaseNet may be used in the training of diffusion models [48] in order to generate high fidelity omics samples for improved model training. Here, omics models may have a significant advantage over computer vision models in several ways. Primarily, the input size is smaller. The input size of computer vision models could be an order of magnitude larger than DiseaseNet. Second, the features may not be consistent in a computer vision model as the subject content and placement of objects in images changes from picture to picture creating a very high degree of variability within image datasets. In DiseaseNet, the same input feature always represents the same CpG island. The increased feature complexity and input feature size implies that the training sets for image recognition tasks must be vastly larger than those used to train omics models. However, larger omics datasets would still benefit models trained on them, if they are well constructed. Thus, while diffusion models in computer vision may need a large of examples to generate reliable outputs, omics diffusion models may need far less, due to the lower complexity of the inputs. While this remains to be seen and no diffusion model based on omics data has been produced as of the publication of this report, we remain optimistic that these models will be the future of model building for the biological and medical diagnostic space. Future works could also exploit advanced deep learning methods, including graph representation learning and heterogeneous information network models. By exploring these avenues, a robust framework for the continued development and improvisation of disease diagnosis tools could be established, ensuring adaptability to emerging methodologies and technologies in the field.

## Conclusions

DiseaseNet, our innovative model designed to leverage useful information encoded in cancer methylation data, demonstrated robust performance in classifying non-communicable diseases (NCDs), including arthritis, asthma, and SCZ. With an overall accuracy (average F-measure) of 94.5%, the performance of DiseaseNet surpassed that of the frequently used machine learning models on the same NCD dataset. Comparison of deep learning models with and without transfer learning demonstrated the advantages of transfer learning in disease classification, specifically, the capability of such models to perform cross-disease knowledge transfer to achieve more robust disease diagnosis. Beyond classification, DiseaseNet exemplifies synergies between cancer and non-cancer NCDs at the epigenetic level. Our study could spur further research on development of unified disease models and on unraveling and understanding shared molecular features among diseases.

### Supplementary Information

**Additional file 1: Table 1** Count of samples by disease class (row) and sample source (column). **Table 2** TCAV output for Arthritis. The rows are the sample tissue source and the columns give, in order, the TCAV score, the TCAV score of randomly chosen samples against those in the target tissue source, and the P-value of the TCAV score. A P-value of 0.005 or less was deemed statistically significant. **Table 3** TCAV output for Asthma. The rows are the sample tissue source and the columns give, in order, the TCAV score, the TCAV score of randomly chosen samples against those in the target tissue source, and the P-value of the TCAV score. A P-value of 0.005 or less was deemed statistically significant. **Table 4** TCAV output for Schizophrenia (SCZ). The rows are the sample tissue source and the columns give, in order, the TCAV score, the TCAV score of randomly chosen samples against those in the target tissue source, and the P-value of the TCAV score. A P-value of 0.005 or less was deemed statistically significant. **Table 5** TCAV output for Normal (NORM). The rows are the sample tissue source and the columns give, in order, the TCAV score, the TCAV score of randomly chosen samples against those in the target tissue source, and the P-value of the TCAV score. A P-value of 0.005 or less was deemed statistically significant.

## Data Availability

The software and associated datasets are available at https://github.com/Sgore83/DiseaseNet. All other datasets are provided with the article and supplementary materials. We also declare that no biomolecular data (e.g. proteomics data and protein sequences, DNA and RNA sequences, genetic polymorphisms, linked genotype and phenotype data, macromolecular structure, gene expression data, crystallographic data for small molecules) were generated from this work. The GEO datasets used in this study were obtained from NCBI GEO repository (https://www.ncbi.nlm.nih.gov/geo/), with accession numbers GSE36054, GSE41169, GSE56553, GSE69270, GSE71841, GSE89251, GSE99863, GSE111942, GSE121192, GSE152027, GSE174422.
